# Cost-effectiveness of a gestational age metabolic algorithm for preterm and small-for-gestational-age classification

**DOI:** 10.1016/j.ajogmf.2020.100279

**Published:** 2021-01

**Authors:** Kathryn Coyle, Amanda My Linh Quan, Lindsay A. Wilson, Steven Hawken, A. Brianne Bota, Doug Coyle, Jeffrey C. Murray, Kumanan Wilson

**Affiliations:** a Institute of Environment, Health and Societies, Brunel University London, Kingston Lane, Uxbridge, Middlesex, United Kingdom; b Clinical Epidemiology Program, Ottawa Hospital Research Institute, Ottawa, Ontario, Canada; c Faculty of Medicine, School of Epidemiology and Public Health, University of Ottawa, Ottawa, Ontario, Canada; d Department of Pediatrics, University of Iowa, Iowa City, IA; e Bruyère Research Institute, Ottawa, Ontario, Canada

**Keywords:** cost-effectiveness, economic evaluation, metabolomics, preterm birth, small for gestational age

## Abstract

**Background:**

Preterm birth complications are the leading cause of death among children under 5 years of age, and this imposes a heavy burden on healthcare and social systems, particularly in low- and middle-income countries where reliable estimates of gestational age may be difficult to obtain. Metabolic analyte data can aid in accurately estimating gestational age. However, important costs are associated with this approach, which are related to the collection and analysis of newborn samples, and its cost-effectiveness has yet to be determined.

**Objective:**

This study aimed to evaluate the cost-effectiveness of an internationally validated gestational age estimation algorithm based on neonatal blood spot metabolite data in combination with clinical and demographic variables (birthweight, sex, and multiple birth status) compared with a basic algorithm that uses only clinical and demographic variables in classifying infants as preterm or term (using a 37-week dichotomous preterm or term classification) and determining gestational age.

**Study Design:**

The cost per correctly classified preterm infant and per correctly classified small-for-gestational-age infant for the metabolic algorithm vs the basic algorithm were estimated with data from an implementation study in Bangladesh.

**Results:**

Over 1 year, the metabolic algorithm correctly classified an average of 8.7 (95% confidence interval, 1.3–14.7) additional preterm infants and 145.3 (95% confidence interval, 128.0–164.7) additional small-for-gestational-age infants per 1323 infants screened compared with the basic algorithm using only clinical and demographic variables. The incremental annual cost of adopting the metabolic algorithm was $100,031 (95% confidence interval, $86,354–$115,725). If setup costs were included, the cost was $120,496 (95% confidence interval, $106,322–$136,656). Compared with the basic algorithm, the incremental cost per preterm infant correctly classified by the metabolic algorithm is $11,542 ($13,903 with setup), and the incremental cost per small-for-gestational-age infant is $688 ($829 with setup).

**Conclusion:**

This research quantifies the cost per detection of preterm or small-for-gestational-age infant in the implementation of a newborn screening program to aid in improved classification of preterm and, in particular, small-for-gestational-age infants in low- and middle-income countries.

**AJOG MFM at a Glance**

**Why was this study conducted?**

A cost analysis of using metabolic algorithms to determine the rates of preterm and small-for-gestational-age (SGA) births has not yet been performed.

**Key findings**

Compared with the basic algorithm that uses only birthweight (BW), sex, and multiple birth status, the implementation of a metabolic algorithm using blood spots collected from newborns to improve the classification of SGA and preterm infants identifies preterm births and SGA infants at a cost of $11,542 ($13,903 with setup) and $688 ($829 with setup), respectively**.** Testing only those infants within the BW range of 2500 to 3600 g has the lowest cost per identified infant.

**What does this add to what is known?**

The incremental annual cost of adopting the metabolic algorithm was $100,031 (95% confidence interval [CI], $86,354–$115,725). If setup costs were included, the cost was $120,496 (95% CI, $106,322–$136,656).

## Introduction

Each year, an estimated 15 million infants are born preterm (before 37 weeks of gestation). Preterm birth complications are the leading cause of death among children under 5 years of age, and this imposes a heavy burden on healthcare and social systems. Small-for-gestational-age (SGA) infants also face considerable morbidity and mortality risks^1^ such as increased risk of increased economic burden owing to healthcare costs or lack of achieving neurodevelopment potential.^2^ The economic cost associated with preterm infants is estimated to be 10 times more than that of term infants, and there is also an associated loss in economic productivity.^3^, ^4^, ^5^, ^6^ Low- and middle-income countries (LMICs) are especially burdened as reliable estimates of gestational age (GA) may be difficult to obtain, making it challenging to distinguish between preterm and term low birthweight (BW) infants, provide appropriate maternal and neonatal care, anticipate complications, or guide preventive measures. Feasible, effective, and cost-effective interventions for decreasing neonatal morbidity in LMICs have been highlighted as a priority by the World Health Organization.^7^ However, for these interventions to be effective, accurate estimates of GA both prenatally and at birth are needed.

Established newborn screening (NBS) programs operating in many regions worldwide yield metabolic analyte data that can aid accurate estimation of GA.^8^ Several groups have developed algorithms that can accurately estimate GA to within ±1 to 2 weeks of ultrasound-validated GA using metabolic data combined with clinical and demographic variables.^10^, ^11^, ^12^, ^9^ These algorithms have also demonstrated greater accuracy in estimating GA than algorithms that solely use clinical and demographic variables.^13^ To our knowledge, no prior study has investigated the cost and cost-effectiveness of using metabolic algorithms compared with algorithms that use clinical information alone (ie, BW, sex, and multiple birth status) to determine rates of preterm and SGA births. To address this knowledge gap, we conducted a cost-effectiveness analysis comparing an internationally validated GA estimation algorithm based on neonatal blood spot metabolite data in combination with sex, BW, and multiple birth status (hereinafter referred to as the metabolic algorithm)^14^ with an algorithm that uses only BW, sex, and multiple birth status (hereinafter referred to as the basic algorithm) in classifying infants as preterm or term (using a 37-week dichotomous preterm or term classification) and determining GA.

## Materials and Methods

### Decision problem

The study was designed to address a specific decision problem: whether a program adopting a GA estimation algorithm that can be used to determine preterm birth based on metabolic analyte data derived from newborn blood spots should be funded within LMICs.^5^ This also permits the identification of SGA infants, defined as those weighing below the 10th percentile for weight at a given GA. Preterm birth and SGA are qualitatively distinct and therefore not possible to combine into a single outcome through a weighting approach. Given the uncertainty over the long-term implications of both outcomes, cost-effectiveness analyses were conducted for both outcomes rather than estimating outcomes in terms of hybrid measures, such as quality-adjusted life year (QALY, where a QALY of 1 is equivalent to living 1 year in full health) or disability-adjusted life year (DALY, defined as the sum of years of productive life lost because of disability). The analysis adopted a governmental perspective, with outcome assessed as number of preterm and SGA births correctly identified.

Initial analysis compared both preterm birth and SGA classifications by using the metabolic algorithm with classification using the basic algorithm. Further analysis specific to preterm birth classifications involved a sequential analysis comparing restricted use of the metabolic algorithm with subsets of the population based on BW. This was based on 2 factors intended to optimize the algorithm: the higher sensitivity with respect to the detection of preterm birth using the basic algorithm in low BW neonates and the low rate of preterm birth in high BW neonates.

### Overview

#### Overview of implementation study and algorithm

The results of the implementation study and further details of the study and the algorithms used for this analysis have been published previously.^12^^,^^14^ Briefly, newborn dried blood spot (DBS) heel prick and cord blood samples were collected in Matlab, Bangladesh, and sent to the Newborn Screening Ontario in Ottawa, Canada. DBS samples were analyzed for the following metabolites: hemoglobin profiles; 17α-hydroxyprogesterone; thyroid-stimulating hormone; immunoreactive trypsinogen; a panel of 12 amino acids and 31 acylcarnitines; T-cell receptor excision circles; biotinidase activity; and galactose-1-phosphate uridylyltransferase activity. The metabolic algorithm combined both clinical data (infant sex, multiple birth status [yes or no], BW, newborn screening analytes, and pairwise interactions) and analyte data. The basic algorithm was modeled as fully and advantageously as possible (ie, BW was splined to allow for nonlinearity, and pairwise interactions were modeled according to BW, sex, and multiple gestations) but contained only locally available clinical data ( infant sex, BW, and multiple birth status [yes or no]).

#### Validation of the metabolic algorithm

In preparation for modeling, newborn screening analytes were Winsorized using an adapted “Tukey fence” approach.^15^ Analyte levels and newborn BWs were standardized to have a mean of 0 and a standard deviation of 1 by subtracting the mean for each analyte and dividing by the standard deviation in each cohort. This affected normalizing analyte and BW results for local factors while preserving relative covariation of analytes and BWs across the spectrum of observed GA. Model performance was externally validated by comparing the estimated GA to the actual ultrasound-validated GA of each participating infant. Performance characteristics for estimating GA across dichotomous GA threshold (≥37 vs <37 weeks of gestation) were evaluated using area under the receiver operator curve from a binary logistic regression model.

**Figure 1:**
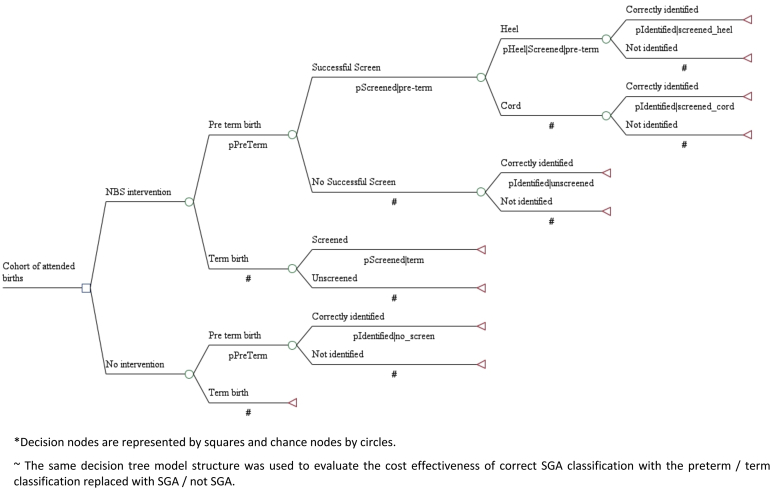
Simplified decision tree Decision nodes are represented by *squares* and chance nodes by *circles*. The same decision tree model structure was used to evaluate the cost-effectiveness of correct SGA classification with the preterm or term classification replaced with SGA or with SGA. *SGA*, small for gestational age. *Coyle et al. Cost analysis of a gestational age algorithm. AJOG MFM 2021.*

To assess the cost-effectiveness of the 2 GA estimation methods, we developed a decision and analytical model in Excel, which took the form of a decision tree (Figure 1). This was used to project the outcomes and cost-effectiveness of implementing a GA estimation algorithm for preterm and SGA classifications. The program mirrored that of our noninterventional validation study in Matlab, Bangladesh.^14^ Specifically, heel prick or cord blood sample collection, newborn screening analysis, and metabolic and GA dating were included. Under the intervention, the true classification of preterm and SGA is based on ultrasound. Effectiveness and cost data were collected during the study (October 2016–July 2018) but were reported as annual outcomes to facilitate interpretability and potential scale-up of the program. Costs in Canadian dollars (CADs) were converted to US dollars (USD) using the respective annual conversion rates (1 CAD=0.75483 USD in 2016; 1 CAD=0.77006 USD in 2017; and 1 CAD=0.77178 USD in 2018).^16^

### Effectiveness data

Outcome data collected for our validation study in Matlab, Bangladesh, were used to determine the effectiveness for this study. The results of the study have been published elsewhere,^14^ but in brief, our study demonstrated that, in a cohort of 1069 newborns, our GA estimation model could accurately estimate GA to within 2 weeks of ultrasound-validated GA 94% of the time. In the current study, the outcomes of analysis were the number of screened infants who were correctly identified as preterm and the number correctly identified as SGA by each GA estimation method based on INTERGROWTH-21st fetal growth standards. For an infant to be correctly identified as preterm, the screening method must accurately classify them as preterm within 1 week of a 37-week dichotomous preterm or term classification (ie, to no more than 38 weeks predicted GA). For correct SGA classification, the SGA classification based on the algorithm estimates must concur with the classification based on the ultrasound-assessed GA. Currently available international classification standards apply only to infants over 32 weeks of gestation.^17^

For the initial analyses, we identified the number of preterm and SGA births correctly classified by the metabolic algorithm compared with the basic algorithm for the entire population of neonates. For the secondary analysis, we identified outcomes with respect to the classification of preterm birth for different ranges of BWs.

### Cost data

A governmental perspective was employed; thus, only costs attributed to the health system were included. The costs are reflective of the costs incurred for the setup and ongoing implementation of the validation study. Patient and research costs were excluded. The costs can be classified into 3 broad categories of one-time setup costs, annual site-specific ongoing costs, and costs per infant screened. The one-time setup costs are those associated with establishing the program at a new site, which will only be incurred when a new site is added to the program. The site-specific ongoing costs are the costs associated with running the program at a single site for 1 year with the assumption that the site is comparable with the validation study site. Both the setup costs and site-specific ongoing costs are consistent across all analyses with the assumption that the analyses are based on a single site and are independent of volume. The costs per infant screened are the variable costs of the program that increase directly in proportion to the number of infants screened. As the number of infants tested increases, the total cost increases, although the average cost per infant screened decreases.

### Analysis

Analysis was presented as both a deterministic analysis and a probabilistic analysis; the latter involved a combined nonparametric bootstrapping technique and Monte Carlo simulation. Uncertainty in costs was incorporated through a gamma distribution with standard errors estimated at 10% of the mean value, and clinical parameters were captured through the bootstrap procedure using the clinical data. Overall, 5000 replications were drawn, which was sufficient to obtain stable estimates of costs and effects. Results are presented as mean values with uncertainty represented by 95% confidence intervals (CIs) generated by bias-corrected bootstrapping.

For the initial analyses, cost-effectiveness was assessed as the difference in costs between the metabolic algorithm and the basic algorithm divided by the increased number of births correctly classified with respect to first preterm and then SGA. For the secondary analysis, we conducted a sequential analysis to identify the optimal range for screening with respect to preterm classification. The reporting of the sequential analysis focused on different ranges with a lower limit of testing set at 2500 g and an upper limit ranging from 2600 to 5000 g—all other ranges were subject to dominance (both costlier and no more effective than other ranges).

## Results

After adjusting the results of the validation study to a 1-year period, 1323 infants were tested, and 1015 of the resulting samples (325 by heel prick and 690 by cord blood) were suitable for analysis (ie, adequate blood quantity and sample quality) and included in this analysis. According to ultrasound determination, 92 infants (9.1%) with satisfactory samples were preterm (GA<37 weeks), and 249 infants (24.6%) were SGA.

Based on the combined effectiveness of the heel and cord blood samples, the metabolic algorithm would correctly identify 69.6% (n=64.0 per annum [95% CI, 52.0–76.7], 6.3% of infants) of the 92 preterm infants as preterm within a 1-week precision. In contrast, the basic algorithm correctly identified 60.1% (n=55.3 per annum [95% CI, 44.7–68.0], 5.4% of all infants) of the preterm infants. With respect to accurate classification of SGA infants, the metabolic algorithm correctly classified 71.9% (n=179.3; 95% CI, 159.3–200.0) of true SGA infants compared with the basic algorithm, which correctly classified 13.6% (n=34.0; 95% CI, 24.7–43.3).

### Costs of screening by gestational age algorithm study

Table 1 summarizes the costs of adopting the metabolic algorithm-screening method. Annual cost was estimated to be $120,496, which includes one-time setup costs, annual site-specific ongoing costs, and costs per infant screened. Setup costs were $9396 incurred at the site for administration and training of personnel and $11,069 incurred in Canada for setting up the processes for receiving and testing the samples, provision of results, and personnel training. In addition, 63% of ongoing annual cost was incurred at the site and included salaries for individuals overseeing and administering the program. The remaining 37% was incurred in Canada ($13,552) and included laboratory salaries, managerial oversight, and shipping. The total ongoing annual costs of the program were estimated at $100,031.

**Table 1:** Cost of adopting the metabolic GA estimation algorithm

Variable	Costs in USD (95% CI)
One-time setup costs	$20,465.00 ($16,824.00–$25,046.00)
Ongoing costs	$36,259.00 ($29,662.00–$44,052.00)
Costs per sample	$48.19 ($39.13–$58.87)
Total annual cost excluding setup (based on all patients screened using the algorithm)	$100,031.00 ($86,354.00–$115,725.00)
Total annual cost including setup (based on all patients screened using the algorithm)	$120,496.00 ($106,322.00–$136,656.00)

*CI*, confidence interval; *USD*, United States dollar.

*Coyle et al. Cost analysis of a gestational age algorithm. AJOG MFM 2021.*

### Cost-effectiveness: initial analysis

Implementation of screening using the metabolic algorithm would lead to an additional 8.7 preterm infants (95% CI, 1.33–14.67) correctly identified annually compared with the basic algorithm (Table 2). The incremental annual cost of the metabolic algorithm was $100,031 (95% CI, $86,354–$115,725; $120,496 [95% CI, $106,322–$136,656] including setup costs). Thus, the incremental cost per preterm infant correctly identified was $11,542 ($13,903 with setup).

**Table 2:** Results of gestational age estimation methods for all infants

Variable	Basic algorithm (no screening)	Metabolic algorithm
Number of infants tested per y	—	1323
Number of suitable samples per y	—	1015
Total annual cost excluding setup	—	$100,031
Total annual cost including setup	—	$120,496
Preterm infants		
Number of preterm infants as determined by ultrasound	92	92
Percentage of preterm infants correctly identified (to within 1-wk precision)	60.1	69.6
Number of preterm infants correctly identified (to within 1-wk precision)	55.3	64.0
Additional preterm infants identified through the metabolic algorithm	—	8.7
Incremental cost per preterm infant identified (excluding setup)	—	$11,542
Incremental cost per preterm infant identified (including setup)	—	$13,903
SGA infants		
Number of SGA infants as determined by ultrasound	249.3	249.3
Percentage of SGA infants correctly identified	13.6	71.9
Number of SGA infants correctly identified	34.0	179.3
Additional SGA infants identified through the metabolic algorithm	—	145.3
Incremental cost per SGA infant identified (excluding setup)	—	$688
Incremental cost per SGA identified (including setup)	—	$829

Costs in US dollars.

*SGA*, small for gestational age.

*Coyle et al. Cost analysis of a gestational age algorithm. AJOG MFM 2021.*

With respect to the correct classification as SGA, the metabolic algorithm led to an additional 145.3 infants (95% CI, 128.0–164.7) correctly classified compared with the basic algorithm. The incremental costs were the same as the preterm analysis, resulting in an incremental cost per SGA infant correctly classified of $688 ($829 with setup).

**Figure 2:**
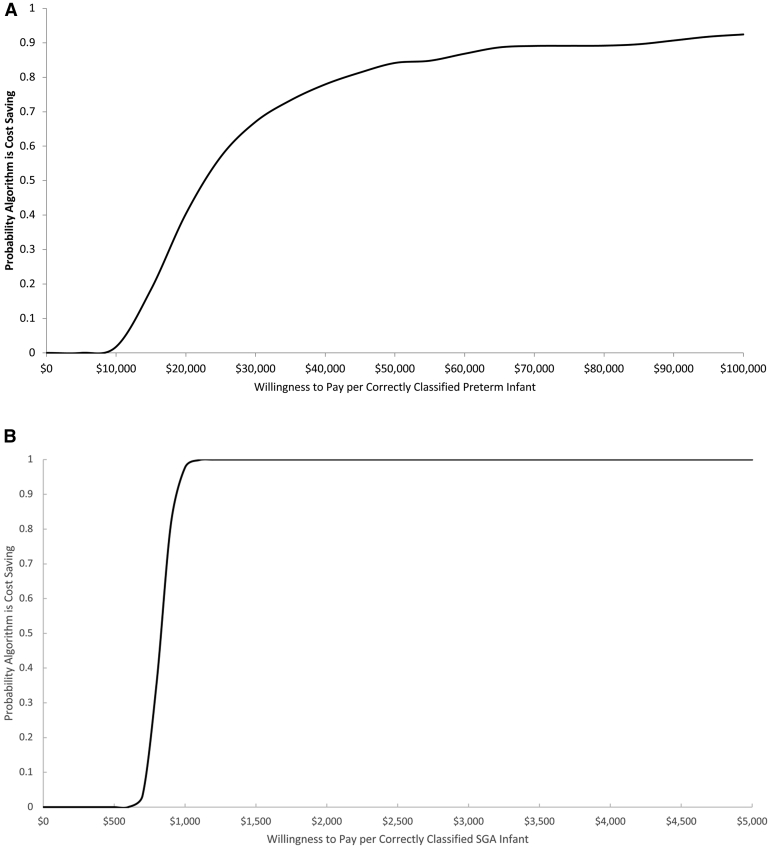
Probability metabolic algorithm **A,** Cost-effective vs basic algorithm at various WTP thresholds per correctly classified preterm infant. **B,** Cost-effective vs basic algorithm at various WTP thresholds per correctly classified small-for-gestational-age infant. *WTP*, willingness to pay. *Coyle et al. Cost analysis of a gestational age algorithm. AJOG MFM 2021.*

A cost-effectiveness acceptability curve was derived to depict the probability that the metabolic algorithm was cost-effective at different threshold values of the willingness to pay (WTP) for a correctly classified preterm infant and for a correctly classified SGA infant (Figure 2). For example, there is a 56% probability that the metabolic algorithm is cost-effective at a WTP of $15,000 and a 100% probability at any WTP for a correctly classified SGA infant value greater than $1100.

### Cost-effectiveness: secondary analysis

**Figure 3:**
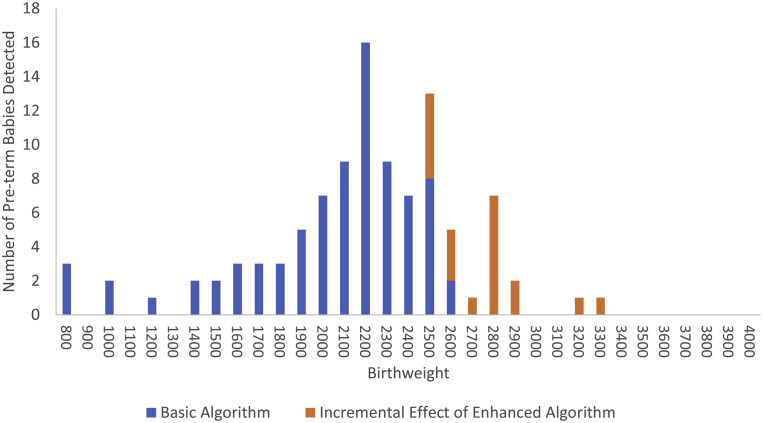
Incremental benefit Incremental benefit of metabolic algorithm vs basic algorithm for preterm-term classifications. *Coyle et al. Cost analysis of a gestational age algorithm. AJOG MFM 2021.*

The use of the metabolic algorithm in the BW cohort below 2500 g and above 3500 g cannot be considered cost-effective as infants under 2500 g are more accurately classified as preterm using the basic algorithm and no infant over 3600 g was preterm (Figure 3). Table 3 provides data on the number of additional preterm infants correctly identified as preterm based on BW ranges from 2500 to 2600 g as an initial range to 2500 to 4000 g as the broadest range.

**Table 3:** Data relating to effectiveness and cost-effectiveness of applying metabolic GA estimation algorithm to different BW cohorts

BW range	Infants tested	Preterm births based on ultrasound (37-wk cutoff), n	Preterm births identified by basic algorithm (<38 wk), n	Number of preterm births identified by metabolic GA algorithm (<38 wk), n	Total cost excluding setup	Total cost including setup	Incremental cost per preterm infant identified vs basic algorithm (excluding setup)	Incremental cost per preterm infant identified vs basic algorithm (including setup)
2500–2600 g	117	14.0	5.3	9.3	$41,911	$62,376	$10,478	$15,594
2500–2700 g	219	16.0	5.3	10.0	$46,810	$67,276	$10,031	$14,416
2500–2800 g	364	29.3	5.3	14.7	$53,803	$74,268	$5765	$7957
2500–2900 g	493	33.3	5.3	16.0	$60,000	$80,465	$5625	$7544
2500–3000 g	689	37.3	5.3	16.0	$69,464	$89,929	$6512	$8431
2500–3100 g	777	37.3	5.3	16.0	$73,693	$94,158	$6909	$8827
2500–3200 g	871	38.7	5.3	17.3	$78,215	$98,680	$6901	$8707
2500–3300 g	951	40.0	5.3	17.3	$82,067	$102,532	$6839	$8544
2500–3400 g	984	40.0	5.3	17.3	$83,658	$104,124	$6972	$8677
2500–3500 g	1039	40.7	5.3	17.3	$86,338	$106,803	$7195	$8900
2500–3600 g	1060	42.0	5.3	17.3	$87,343	$107,808	$7279	$8984
2500–3700 g	1072	42.0	5.3	17.3	$87,929	$108,395	$7327	$9033
2500–3800 g	1078	42.0	5.3	17.3	$88,223	$108,688	$7352	$9057
2500–3900 g	1083	42.0	5.3	17.3	$88,432	$108,897	$7369	$9075
2500–4000 g	1096	42.0	5.3	17.3	$89,060	$109,525	$7422	$9127

*BW*, birthweight; *GA*, gestational age.

*Coyle et al. Cost analysis of a gestational age algorithm. AJOG MFM 2021.*

For the cohort ranging from 2500 to 2600 g, the incremental cost per preterm infant correctly classified by the metabolic algorithm vs the basic algorithm is $10,478 ($15,594 including setup) (Table 4). However, the incremental cost for the cohort from 2500 to 2900 g is $5,625 ($7544 including setup), suggesting that restricting use to 2500 to 2600 g is subject to extended dominance and not optimal. The incremental cost of expanding the range from 2500 to 2900 g to 2500 to 3300 g is estimated at $16,550 per additional preterm infant correctly classified. All alternative ranges, including testing all infants, are subject to dominance or extended dominance.

**Table 4:** Sequential cost utility analysis for different ranges of applying the metabolic GA estimation algorithm

Strategies by GA algorithm use	Costs of applying the metabolic GA estimation algorithm	Preterm infant correctly identified	Incremental cost per preterm infant correctly identified vs basic algorithm	Sequential ICER ($/preterm infant correctly identified)
Nondominated strategies				
No GA algorithm use	—	47.3	—	—
2500–2900 g BW	$60,000	58.0	$5625	$5625
2500–3300 g BW	$82,067	59.3	$6839	$16,550
Dominated therapies				
2500–2600 g BW	$41,911	51.3	$10,478	Subject to extended dominance through no GA algorithm use and 2500–2900 g BW
2500–2700 g BW	$46,810	52.0	$10,031	Subject to extended dominance (as above)
2500–2800 g BW	$58,803	56.6	$5765	Subject to extended dominance (as above)
2500–3000 g BW	$69,464	58.0	$6512	Dominated by 2500–2900 g BW
2500–3100 g BW	$73,693	58.0	$6909	Dominated by 2500–2900 g BW
2500–3200 g BW	$78,215	58.6	$6901	Subject to extended dominance through 2500–2900 g BW and 2500–3300 g BW
2500–3400 g BW	$83,658	59.3	$6972	Dominated by 2500–3300 g BW
2500–3500 g BW	$86,338	59.3	$7195	Dominated by 2500–3300 g BW
2500–3600 g BW	$87,343	59.3	$7279	Dominated by 2500–3300 g BW
2500–3700 g BW	$87,929	59.3	$7327	Dominated by 2500–3300 g BW
2500–3800 g BW	$88,223	59.3	$7352	Dominated by 2500–3300 g BW
2500–3900 g BW	$88,432	59.3	$7369	Dominated by 2500–3300 g BW
2500–4000 g BW	$89,060	59.3	$7422	Dominated by 2500–3300 g BW
All infants	$100,031	59.3	$8336	Dominated by all restricted use strategies

*BW*, birthweight; *GA*, gestational age; *ICER*, incremental cost-effectiveness ratio.

*Coyle et al. Cost analysis of a gestational age algorithm. AJOG MFM 2021.*

## Comment

This study determines the cost-effectiveness of implementing a metabolic GA estimation algorithm compared with standard practice in many LMICs of using only sex, BW, and multiple birth status to determine GA postnatally. The incremental benefits of using the metabolic algorithm vs the basic algorithm, based on effectiveness data from our validation study in Matlab, Bangladesh, included the correct classification of an additional 145.3 SGA infants and 8.7 preterm infants per 1323 infants screened annually (an additional 109.8 SGA and 6.6 preterm infants correctly classified per 1000 live births). It is important to note that our metabolic algorithm was designed to identify exact GA rather than a dichotomous cutoff of preterm and term. Improved detection of preterm births could occur by tailoring the metabolic algorithm for this purpose, but it would be at the cost of the precision of the GA estimate. This would also limit the ability to identify SGA infants across a spectrum of GAs. In this scenario, when all infants are tested, the incremental cost per preterm infant identified by the metabolic algorithm vs the basic algorithm is $13,903 ($11,542 excluding setup). Clinical variables alone are likely sufficient to estimate preterm rates at very low and high BWs, as misclassification rates in these groups would be low. Thus, a parsimonious approach where GA estimation using more complex techniques can be limited to a specific BW range is worth considering if the sole purpose is to establish preterm birth rates. Through further analysis, it was found that only testing neonates within the range of 2500 to 3600 g would make the implementation more cost-effective than testing all infants as infants born weighing <2500 g can be accurately identified as preterm based on the basic algorithm, and there was no preterm infant born weighing above 3600 g. With the data available through our validation study in Matlab, Bangladesh, optimal cost-effectiveness of using our metabolic algorithm appears to be within the BW range of 2500 to 2900 g with an incremental cost per preterm infant identified of $5625 ($7544 including setup).

With respect to the detection of preterm infants, there is the potential for the cost-effectiveness of the metabolic algorithm to be improved if the specification could be refined to identify more preterm infants between 2500 and 3600 g. It should also be noted that, given the nature of the metabolic algorithm and the data it was trained on, there is the potential that 3 times as many additional preterm infants could be identified if all the samples were heel prick samples (assuming effectiveness can be applied) as opposed to 68% of samples being from cord blood. Alternatively, the metabolic algorithm can be further refined, or separate algorithms for each sample type could be developed to optimize effectiveness and cost-effectiveness. In addition, if the cost per infant tested could be reduced, possibly by incorporating on-site testing, the cost-effectiveness would also be improved. It should be noted that the algorithm’s performance may vary among different populations; as more data become available and a plausible performance range can be established, sensitivity analyses should be done.

The results are also limited to the study environment in Matlab. Although we expect many of the costs and properties of the diagnostic test to be similar in other LMICs, further evaluations in other LMICs and concurrent economic evaluations are warranted. Bangladesh has high rates of both preterm birth and SGA.^18^ The SGA rates exceed 30%, so the cost-effectiveness we calculated is driven in part by this high rate. It is also important to note that this study did not take into account the additional benefit of identifying infants with specific conditions, such as congenital hypothyroidism through the NBS process that is the basis of the GA estimation. In our Matlab cohort, infants with specific conditions were identified, and treatment was initiated.^14^

The current study did not follow newborns beyond birth. To better understand the cost of identifying preterm or SGA infants, following a cohort of patients for health service utilization may further inform the economic value of early identification of preterm and SGA infants and allow for the determination of cost per QALY or DALY. Future research should attempt to elucidate these benefits and costs. Evaluating the cost and benefits of implementing ultrasound in LMICs would also be beneficial. Ultrasound is considered the gold standard for estimating GA, but its availability is limited in LMICs. Thus, the economic analysis of ultrasound has predominantly focused on high-resource settings. Although the availability of ultrasound in low-resource settings has increased over time,^19^ other obstacles have been raised in implementing ultrasound, including misuse, overuse, and neglect of conventional methods of care and the ability to maintain equipment and supplies.^20^ Many variables need to be considered before ultrasound is adopted in a low-resource setting. Further research should consider the best approaches in supporting newborn care in low-resource settings from both a cost perspective and a healthcare perspective.

It is difficult to determine what would be an acceptable cost for identifying preterm or SGA status. Currently, interventions for infants in LMICs are based on BW, in part, because of an inability to accurately assess GA.^21^^,^^22^ With improved identification of preterm infants and exact GA, the potential to explore interventions to benefit these infants will be expanded. There are therapeutic benefits to identifying an infant as preterm or SGA (or both) that include expectant management of disorders, such as hypoglycemia or hyperbilirubinemia where failure to initiate therapy can quickly result in even poorer neurodevelopmental outcomes and even death. Preterm infants can be monitored more closely for high-risk outcomes, such as retinopathy of prematurity, intraventricular hemorrhage, or patent ductus arteriosus, all of which are more likely to occur at lower GAs. SGA can also have infectious etiologies that could be noticeable, thereby preventing transmission to other neonates.

Our results suggest that a metabolic approach is efficient in identifying SGA infants. The increased efficiency in identifying SGA infants over preterm infants is because BW is relatively effective at identifying preterm birth but not at determining SGA. BW alone may suggest that an infant is preterm when they may actually be term and small. This is evident in our study as the basic algorithm, which relies heavily on BW, only predicts SGA at a rate of 13.6%. At a population level, if a program screened 1000 infants per annum, the cost per infant would be $104.93 including the setup of the program and $84.46 on an ongoing basis omitting setup costs. We expect that this ongoing cost of approximately $85,000 per year would provide reliable population level estimates of preterm birth and SGA for a jurisdiction.

### Conclusion

This study evaluates the cost-effectivness of a GA algorithm in LMICs. In addition to our algorithm,^14^ 2 other groups in North America^10^^,^^11^ have developed metabolic dating algorithms based on health administrative datasets. Further investigations into the feasibility and cost-effectiveness of metabolic GA assessments will inform governments and global health-funding initiatives into the usefulness of these initiatives in LMICs. Ultimately, decision makers and funders will have to consider the cost of the various interventions compared with their potential benefits when deciding how to allocate scarce resources.
